# Mutations in *pmrB* Confer Cross-Resistance between the LptD Inhibitor POL7080 and Colistin in *Pseudomonas aeruginosa*

**DOI:** 10.1128/AAC.00511-19

**Published:** 2019-08-23

**Authors:** Keith P. Romano, Thulasi Warrier, Bradley E. Poulsen, Phuong H. Nguyen, Alexander R. Loftis, Azin Saebi, Bradley L. Pentelute, Deborah T. Hung

**Affiliations:** a The Broad Institute of MIT and Harvard, Cambridge, Massachusetts, USA; b Department of Molecular Biology, Center for Computational and Integrative Biology, Massachusetts General Hospital, Boston, Massachusetts, USA; c Division of Pulmonary and Critical Care Medicine, Brigham and Women’s Hospital, Boston, Massachusetts, USA; d Department of Genetics, Harvard Medical School, Boston, Massachusetts, USA; e Department of Chemistry, Massachusetts Institute of Technology, Cambridge, Massachusetts, USA

**Keywords:** murepavadin, POL7001, POL7080, *Pseudomonas*, antibiotic resistance, colistin, multidrug resistance, *pmrB*, polymyxins

## Abstract

Pseudomonas aeruginosa is a major bacterial pathogen associated with a rising prevalence of antibiotic resistance. We evaluated the resistance mechanisms of P. aeruginosa against POL7080, a species-specific, first-in-class antibiotic in clinical trials that targets the lipopolysaccharide transport protein LptD. We isolated a series of POL7080-resistant strains with mutations in the two-component sensor gene *pmrB*. Transcriptomic and confocal microscopy studies support a resistance mechanism shared with colistin, involving lipopolysaccharide modifications that mitigate antibiotic cell surface binding.

## Abstract (precis)

Pseudomonas aeruginosa is a major bacterial pathogen associated with a rising prevalence of antibiotic resistance. We evaluated the resistance mechanisms of P. aeruginosa against POL7080, a species-specific, first-in-class antibiotic in clinical trials that targets the lipopolysaccharide transport protein LptD. We isolated a series of POL7080-resistant strains with mutations in the two-component sensor gene *pmrB*.

## TEXT

Pseudomonas aeruginosa and other Gram-negative bacteria are becoming increasingly resistant to current antibiotics and pose a major threat to patients with hospital-acquired infections, compromised immune systems, or chronic pulmonary infections (1–4). Unfortunately, the discovery of new agents targeting Gram-negative bacteria is especially challenging due to an impermeable, lipopolysaccharide (LPS)-laden, outer membrane, in addition to porin mutations and efflux pumps limiting intracellular drug accumulation (5, 6). Among the last-resort antibiotics currently being used to treat severe multidrug-resistant pseudomonal infections is the polymyxin class of cationic antimicrobial peptides, including polymyxin B and colistin (polymyxin E). Recently, the first-in-class antibiotic POL7080, which is currently in phase 3 clinical trials, was reported to have species-specific activity against P. aeruginosa by inhibiting the LPS transport protein LptD (7–9). The discovery of POL7080 (and its analogue POL7001) emerged from extensive chemical modifications of the cationic antimicrobial peptide protegrin-1 (PG-1), in which a beta-hairpin was introduced to create cyclized peptidomimetic analogues (10, 11). The mechanism of action of POL7080 and its analogues differs from that of other cationic antimicrobial peptides in several key ways. Polymyxins and PG-1 interact with LPS and exhibit broad-spectrum antimicrobial activity through self-promoted uptake across the outer membrane, followed by cell lysis through poorly defined mechanisms (12–14). The LptD inhibitors POL7080 and POL7001, however, have been reported to exhibit a nonlytic mechanism of action through LptD inhibition in P. aeruginosa exclusively (7–9).

To investigate the mechanisms of resistance to POL7080 and its analogues, we selected for spontaneously resistant P. aeruginosa PA14 mutants by plating mid-log cultures on lysogeny broth (LB) agar containing 1.6 μg/ml POL7001 (∼4 times the MIC on LB agar; detailed experimental protocols are outlined in the supplemental material). We isolated 6 independent mutants and confirmed their resistance to POL7080, POL7001, and PG-1 by adapting the broth microdilution method described previously (15). All clones were highly resistant to POL7080, with 4-fold to 32-fold MIC shifts relative to PA14 (Table 1), comparable to 4 POL7080-resistant clinical isolates reported previously (16). Because resistant clones were selected in LB, MIC assays were also performed in LB, yielding results comparable to those obtained in Mueller-Hinton broth (MHB) (see Table S1 in the supplemental material). All strains remained susceptible to conventional antipseudomonal antibiotics (Table S2). Whole-genome sequencing revealed 1 or 2 single-nucleotide polymorphisms (SNPs) in each mutant, relative to the wild-type (WT) parent PA14, and all 6 isolates carried a mutation in the common gene *pmrB* (Table 1). PmrB is a histidine kinase and the membrane-bound sensor in the PmrA-PmrB two-component regulatory system in Gram-negative bacteria. In response to low Mg^2+^ levels and periplasmic antimicrobial peptides, PmrB undergoes conformational changes in its histidine kinase and methyl-accepting protein (HAMP) domain, leading to autophosphorylation, phosphoryl group transfer to its cognate response regulator PmrA, and downstream activation of transcriptional programs regulating LPS modification (17). Interestingly, mutations in the PmrA-PmrB system have been implicated in polymyxin resistance through upregulation of the lipid A deacylase PagL and the *arnBCADTEF-ugd* operon, resulting in LPS modifications that reduce polymyxin binding to the cell surface (18–29). Notably, the mutant PA14-*pmrB*_G188S_ contained an amino acid substitution at the same site in the HAMP domain as reported previously for the colistin-resistant clinical strain PA1571-*pmrB*_G188D_, which was isolated from a cystic fibrosis patient and was found to have the PmrB substitution G188D (22). Therefore, we measured colistin activity against all 6 resistant mutants and found cross-resistance, with MIC shifts ranging from 4-fold to 32-fold (Table 1). We also tested the colistin-resistant strain PA1571-*pmrB*_G188D_ with POL7080 and found that it had 8-fold cross-resistance, relative to PA14 (Table 1).

**TABLE 1:** Summary of resistant mutants sequenced after selection with POL7001

Strain	PA14 no.	Gene	SNP(s)	Protein change(s)	Function	MIC (μg/ml) in MHB (fold change)^*a*^			
						POL7001	POL7080	PG-1	Colistin
Wild-type PA14						0.050	0.050	1.3	0.44
PA14-*pmrB*_L172del_	63160	*pmrB*	CGCT506C	L172del^*b*^	Two-component system	1.6 (32)	1.6 (32)	43 (32)	14 (32)
PA14-*pmrB*_G188S_	63160	*pmrB*	G562A	G188S	Two-component system	0.40 (8)	0.20 (4)	11 (8)	1.8 (4)
	21890	*hypo*^*c*^	T932G	V311G	Putative oxidoreductase				
PA14-*pmrB*_V136L_	63160	*pmrB*	G406C	V136L	Two-component system	0.80 (16)	0.40 (8)	>43 (>32)	3.5 (8)
	43080	*vgrG14*	C1325A + C1330G	A442E + H444D	Type VI secretion				
PA14-*pmrB*_T132P_	63160	*pmrB*	A394C	T132P	Two-component system	0.40 (8)	0.40 (8)	>43 (>32)	3.5 (8)
PA14-*pmrB*_R155H_	63160	*pmrB*	G464A	R155H	Two-component system	1.6 (32)	1.6 (32)	>43 (>32)	7.0 (16)
PA14-*pmrB*_A330P_	63160	*pmrB*	G988C	A330P	Two-component system	0.80 (16)	0.80 (16)	>43 (>32)	3.5 (8)
PA1571-*pmrB*_G188D_	63160	*pmrB*	G563A	G188D	Two-component system	0.20 (4)	0.40 (8)	10.8 (8)	>56 (>64)

a The fold change in MIC, relative to PA14, is shown in parentheses.

b In-frame deletion of L172.

c Hypothetical protein.

To confirm that alterations in *pmrB* account for the observed resistance to POL7080 and colistin in PA14, we introduced a copy of the wild-type allele *pmrB*_WT_ and the resistant alleles *pmrB*_L172del_ and *pmrB*_G188S_, under the control of an arabinose promoter, into PA14 at the neutral *att*Tn*7* chromosomal site using the pUC18-derived mini-Tn*7* integration system (30). We also introduced the allele *pmrB*_G188D_, which was reported previously to confer colistin resistance (22), into the wild-type background. MIC assays in the presence of 0.25% (vol/vol) arabinose demonstrated that all three mutated *pmrB* alleles, but not the wild-type allele, conferred POL7080 and colistin resistance (Table 2). Because the PA14-*pmrB*_G188S_ mutant was only modestly resistant to POL7080 in MHB, we also determined MICs in LB, the medium in which the mutants were selected, which confirmed that all mutant alleles conferred POL7080 and colistin resistance (Table S3). Conversely, expression of the *pmrB*_WT_ allele in the resistant PA14-*pmrB*_L172del_ background did not restore POL7080 susceptibility, suggesting that resistant *pmrB* alleles were largely dominant over the wild-type allele.

**TABLE 2:** MICs in MHB with 0.25% arabinose after introduction of second *pmrB* alleles into PA14 and *pmrB*_L172del_ backgrounds

Background strain	*att*Tn*7* allele	MIC (μg/ml) (fold change)^*a*^	
		POL7080	Colistin MIC
PA14		0.05	0.44
PA14	*pmrB*_WT_	0.10 (2)	0.44 (1)
PA14	*pmrB*_L172del_	0.80 (16)	1.8 (4)
PA14	*pmrB*_G188S_	0.10 (2)	0.88 (2)
PA14	*pmrB*_G188D_	0.40 (8)	1.8 (4)
PA14-*pmrB*_L172del_		1.6	7.0
PA14-*pmrB*_L172del_	*pmrB*_WT_	0.8 (0.5)	3.5 (0.5)

a The fold change in MIC, relative to the background strain, is indicated in parentheses.

**FIG 1:**
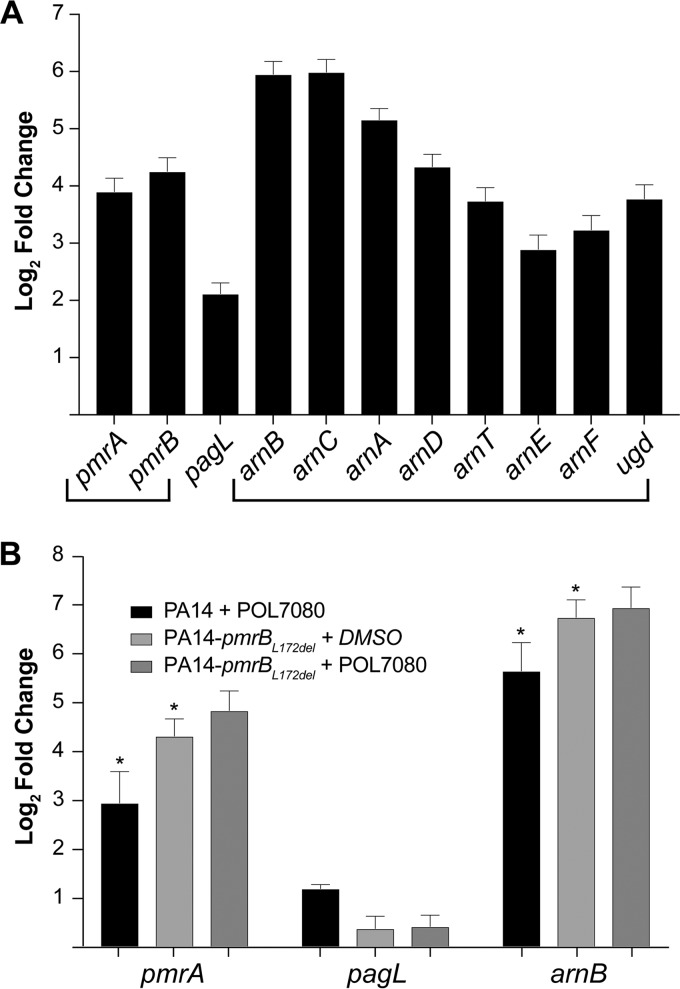
LPS modification genes upregulated in response to POL7001 and POL7080 treatment and constitutively expressed in the resistant PA14-*pmrB*_L172del_ strain. (A) RNA-seq data show log_2_(fold change) in sequencing reads for PA14 after treatment at 37°C for 100 min with POL7001, relative to the vehicle control. Bracketed genes are located within the same operon. Upregulated genes include the *pmrA-pmrB* two-component regulatory system genes, the lipid A deacylase gene *pagL*, and the *arnBCADTEF-ugd* operon. (B) After treatment of PA14 and resistant PA14-*pmrB*_L172del_ cells with POL7080 or dimethyl sulfoxide (DMSO) (control) at 37°C for 100 min, qRT-PCR data show log_2_(fold change) in LPS modification gene transcript levels (normalized to *rpoD* expression), relative to vehicle-treated PA14 cells. In all experiments, error bars represent standard errors of the mean of 3 biological replicates (*n* = 3). The coefficient of variance of raw triplicate measurements ranged from 0.5 to 8.4%. Asterisks indicate paired *t* test *P* values of <0.03 for POL7080-treated PA14 cells versus vehicle-treated PA14-*pmrB*_L172del_ cells.

We next performed whole transcriptome sequencing to investigate the role of *pmrA-pmrB* in response to LptD inhibitors. After extracting total RNA from mid-log PA14 cells treated with 0.2 μg/ml POL7001 (2 times the MIC), we prepared RNA-seq libraries using the RNA TagSeq protocol (31), sequenced samples on an Illumina NextSeq instrument, and analyzed the data using Burrows-Wheeler Aligner (32) for alignment and DESeq2 (33) to determine differential gene expression. We found that LPS modification genes, including *pmrA-pmrB*, the lipid A deacylase gene *pagL*, and the entire *arnBCADTEF-ugd* operon, which is responsible for adding 4-amino-4-deoxy-l-arabinose (l-Ara4N) to lipid A, were significantly upregulated in response to POL7001 (Fig. 1A). The aminotransferase gene *arnB*, coding for the protein that catalyzes the final step in l-Ara4N addition, was among the most highly upregulated genes in the entire data set. We confirmed these findings with quantitative reverse transcription-PCR (qRT-PCR) after treating mid-log PA14 or resistant PA14-*pmrB*_L172del_ cells with either POL7080 or DMSO (control) (Fig. 1B). Relative to the control, POL7080 significantly induced *pmrA*, *arnB*, and *pagL* expression in PA14 cells. Notably, *arnB* and *pmrA* transcript levels in untreated PA14-*pmrB*_L172del_ cells exceeded those in POL7080-treated PA14 cells. Together, these data reveal that a signature transcriptional program involving key LPS modification genes is constitutively upregulated in the resistant mutant PA14-*pmrB*_L172del_. These results reveal a cellular response to LptD inhibitors that mirrors the previously reported response to polymyxins, and they suggest a shared mechanism by which *pmrB* mutations confer cross-resistance between POL7080 and colistin.

**FIG 2:**
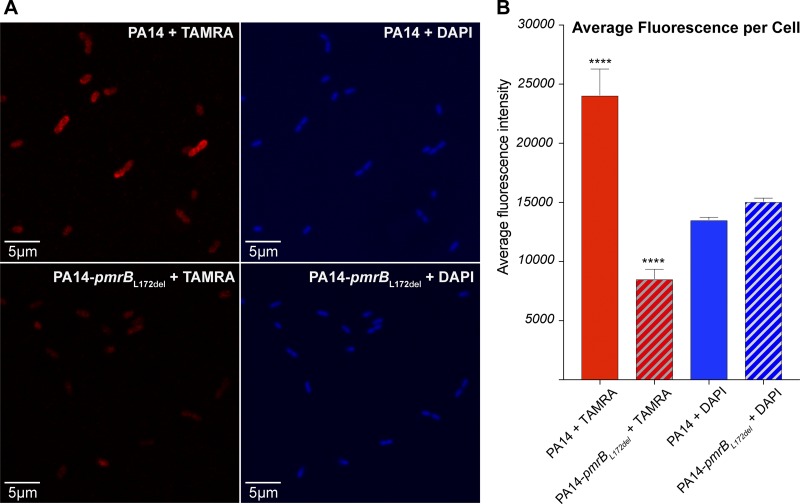
Differential uptake of TAMRA-L27-11 by PA14 cells versus PA14-pmrB_L172del_ cells. (A) Red-field (left) and blue-field (right) confocal microscopy images of PA14 cells (top) and PA14-pmrB_L172del_ cells (bottom) show reduced TAMRA-L27-11 uptake in PA14-*pmrB*_L172del_ cells, relative to PA14 cells. All cells were DAPI stained after treatment with 1.4 μg/ml TAMRA-L27-11 for 120 min. (B) Average fluorescence intensities of TAMRA (red bars) and DAPI (blue bars) were calculated for PA14 cells (solid bars) (*n* = 22 cells) and resistant PA14-*pmrB*_L172del_ cells (hatched bars) (*n* = 21 cells) using ImageJ software. The figure depicts a representative replicate from 3 independent experiments. Error bars represent standard errors of the mean, and asterisks indicate unpaired *t* test *P* values of <0.0001 for PA14 cells versus PA14-*pmrB*_L172del_ cells after TAMRA-L27-11 treatment.

Mutations in *pmrB* are known to drive polymyxin resistance by l-Ara4N addition to LPS, thereby reducing drug binding to the cell surface (18–29). Therefore, we investigated whether *pmrB* mutations might mitigate POL7080 binding to the cell surface. We synthesized tetramethylrhodamine (TAMRA)-L27-11 (Fig. S1), a red fluorescent analogue of POL7080 with retained inhibitory activity (Table S4), to probe for differential uptake in PA14-*pmrB*_L172del_ cells, relative to PA14 cells, using confocal microscopy. After treatment of mid-log PA14 or PA14-*pmrB*_L172del_ cells with 1.4 μg/ml TAMRA-L27-11, the cells were washed, fixed with 4% paraformaldehyde, and stained with 4′,6-diamidino-2-phenylindole (DAPI) for nucleic acid visualization. Red-field and blue-field confocal microscopy showed comparable DAPI staining but >3-fold reduction in TAMRA-L27-11 uptake in PA14-*pmrB*_L172del_ cells, relative to PA14 cells (Fig. 2), indicating less efficient drug binding at the cell surface of the resistant mutant.

In summary, we report a series of *pmrB* mutations that confer high-level resistance to POL7080 and moderate cross-resistance to colistin. Expression analysis and confocal microscopy data support a resistance mechanism in which *pmrB* mutations upregulate the *arnBCADTEF-ugd* operon. These data align well with known mechanisms of resistance to polymyxins, in which upregulation of the *arn* operon has been shown to result in LPS modification with l-Ara4N reducing drug binding to the cell surface (21–23). Altogether, our findings suggest that preexisting colistin resistance may limit the utility of POL7080 in a subset of highly resistant P. aeruginosa cases and exposure to POL7080, if it is successfully developed, could inadvertently drive cross-resistance to colistin and other polymyxins.

## Supplementary Material

Supplemental file 1
