# Supplementary material for: Mutations in pmrB Confer Cross-Resistance between the LptD Inhibitor POL7080 and Colistin in Pseudomonas aeruginosa
Source: Antimicrob Agents Chemother. 2019 Aug 23;63(9):e00511-19. doi: 10.1128/AAC.00511-19 (PMC6709506; doi:10.1128/AAC.00511-19)
Supplement: Supplemental file 1 [file AAC.00511-19-s0001.pdf]

Supplemental Materials

Selecting for POL7001 resistant mutants

PA14 (*Pseudomonas aeruginosa* UCBPP-PA14) cells were grown to mid-log phase (OD_{600} 0.4–0.6) in Lysogeny Broth (LB). Cells were pelleted at room temperature with centrifugation (5000g, 10 minutes) and resuspended in LB to yield $1x10^9$ CFU/mL (OD_{600} 1.0). ~10^8 CFU were plated onto agar plates containing 1.6μg/mL POL7001 (~4X the MIC on LB agar). Six resistant mutants were isolated after incubation at 37°C for 16 hours, which were further grown in 5mL LB with 0.3μg/mL POL7001. Total DNA was extracted using the DNeasy Blood and Tissue Kit (Qiagen, Hilden Germany), and subsequently quantified using the High Sensitivity Quant-iT dsDNA Assay Kit (Thermo Fisher Scientific, Waltham MA). Indexed paired-end libraries were generated using the Nextera XT DNA library preparation kit (Illumina, San Diego CA). 25μL of index DNA libraries were mixed with 15μL Agencourt AMPure XP beads (Beckman Coulter, Pasadena CA) in a 0.5mL deep well block (Thermo Fisher Scientific, Waltham MA), and after equilibrating on a 96S Super Magnet (Alpaqua Engineering, Beverly MA) for 2 minutes, the supernatants were removed. Samples were twice washed with 200μL of 80% (v/v) ethanol, and after drying on the magnet stand for 15 minutes, purified DNA was eluted with 25μL water. DNA quality and quantity were determined using the D5000 ScreenTape with the 2200 TapeStation (Agilent, Santa Clara CA). Samples were diluted to a final concentration of 6.8ng/μL, and pooled for sequencing on the MiSeq instrument (Illumina, San Diego CA). Annotated reference genomes were obtained from www.pseudomonas.com(1). Illumina reads were mapped to the PA14 genome and single nucleotide polymorphisms were identified using the Pilon program previously reported(2). All SNPs, including the *pmrB* mutation in the clinical isolate PA1571-$pmrB_{G188D}$, were subsequently confirmed by Sanger sequencing.

MIC determination

All MIC experiments were conducted in biological triplicate in 384-well microplates (Nunc 384-well clear polystyrene plates, Thermo Fisher Scientific, Waltham MA) using the standard microdilution broth method adapted from previously published methods in Nature Protocols(3). For strains containing a second *pmrB* allele

at the attTn7 site, cultures were grown in the presence of 0.25% (v/v) arabinose. For colistin, meropenem, piperacillin, and ceftazidime, 2-fold serial dilutions were performed in water at 200X the desired assay concentrations, after which samples were diluted 1:100 into LB or MHB. For POL7001, POL7080, PG-1 and TAMRA-L27-11, 2-fold serial dilutions were performed in dimethyl sulfoxide (DMSO) at 200X the desired assay concentrations, after which samples were diluted 1:100 into LB or MHB. 30μL of serially diluted drug solutions were mixed with equal volume mid-log bacterial culture to yield final conditions of $5x10^5$ CFU/mL bacteria (OD_{600} 1.0 = 10^9 CFU/mL based on plate counts) with various drug concentrations. The final DMSO concentration did not exceed 0.5% (v/v). Microplates were incubated in a humidity chamber without shaking for 16 hours at 37°C, after which time the OD_{600} was measured in the Spark Multimode Reader (Tecan, Männedorf Switzerland). The clinical strain PA1571-*pmrB*$_{G188D}$, previously reported to contain the PmrB substitution G188D(4), displayed a growth delay and was thus allowed to grow for 30 hours in a humidity chamber without shaking. Data were plotted and analyzed using GraphPad Prism8 software to determine the MICs, defined as the minimal drug concentrations required for complete inhibition of bacterial growth. **Table S1** summarizes the MIC data generated for PA14, the six POL7080-resistant mutants, and the clinical strain PA1571-*pmrB*$_{G188D}$ in both LB and MHB. **Table S2** summarizes the activities of other conventional antibiotics against these strains. **Table S3** contains the MIC data in LB with 0.25% arabinose after introducing a copy of the mutant *pmrB* alleles at the attTn7 chromosomal site. **Table S4** shows the activity of TAMRA-L27-11 against PA14 and the resistant PA14-*pmrB*$_{L172del}$ mutant.

Table S1: MICs against resistant mutants in MHB and LB. The fold-change relative to PA14 is shown in parentheses.

	**MIC (μg/mL) in MHB**				**MIC (μg/mL) in LB**			
**Strain**	**POL7001**	**POL7080**	**PG-1**	**Colistin**	**POL7001**	**POL7080**	**PG-1**	**Colistin**
Wildtype PA14	0.050	0.050	1.3	0.44	0.10	0.10	2.7	0.88
PA14-***pmrB***$_{L172del}$	1.6 (32)	1.6 (32)	43 (32)	14 (32)	1.6 (16)	3.2 (32)	>43 (>16)	7.0 (8)
PA14-***pmrB***$_{G188S}$	0.40 (8)	0.20 (4)	11 (8)	1.8 (4)	0.80 (8)	0.80 (8)	>43 (>16)	3.5 (4)
PA14-***pmrB***$_{V136L}$	0.80 (16)	0.40 (8)	>43 (>32)	3.5 (8)	1.6 (16)	1.6 (16)	>43 (>16)	3.5 (4)
PA14-***pmrB***$_{T132P}$	0.40 (8)	0.40 (8)	>43 (>32)	3.5 (8)	1.6 (16)	1.6 (16)	>43 (>16)	3.5 (4)
PA14-***pmrB***$_{R155H}$	1.6 (32)	1.6 (32)	>43 (>32)	7.0 (16)	1.6 (16)	3.2 (32)	>43 (>16)	3.5 (4)
PA14-***pmrB***$_{A330P}$	0.80 (16)	0.80 (16)	>43 (>32)	3.5 (8)	1.6 (16)	3.2 (32)	>43 (>16)	3.5 (4)
PA1571-***pmrB***$_{G188D}$	0.20 (4)	0.40 (8)	10.8 (8)	>56 (>64)	0.40 (4)	0.80 (8)	22 (8)	>56 (>64)

**Table S2: MICs of conventional anti-pseudomonal antibiotics against PA14 and resistant mutants in MHB and LB. Fold-change relative to wildtype is shown in parentheses.**						
	**MIC (μg/mL) in MHB**			**MIC (μg/mL) in LB**		
**Strain**	**Piperacillin**	**Ceftazidime**	**Meropenem**	**Piperacillin**	**Ceftazidime**	**Meropenem**
Wildtype PA14	2.7	2.7	0.27	2.7	2.7	0.55
PA14-***pmrB**$_{L172del}$*	1.3 (0.5)	2.7 (1)	0.27 (1)	1.3 (0.5)	2.7 (1)	0.55 (1)
PA14-***pmrB**$_{G188S}$*	5.4 (2)	5.4 (2)	0.27 (1)	5.4 (2)	2.7 (1)	0.55 (1)
PA14-***pmrB**$_{V136L}$*	2.7 (1)	5.4 (2)	0.27 (1)	2.7 (1)	2.7 (1)	0.55 (1)
PA14-***pmrB**$_{T132P}$*	5.4 (2)	5.4 (2)	0.27 (1)	5.4 (2)	2.7 (1)	1.1 (2)
PA14-***pmrB**$_{R155H}$*	2.7 (1)	5.4 (2)	0.55 (2)	2.7 (1)	2.7 (1)	1.1 (2)
PA14-***pmrB**$_{A330P}$*	2.7 (1)	5.4 (2)	0.27 (1)	2.7 (1)	2.7 (1)	0.55 (1)
PA1571-***pmrB**$_{G188D}$*	>43 (>16)	>43 (>16)	>4.4 (>16)	>43.7 (>16)	>43.7 (>16)	4.4 (>8)

**Table S3: MICs in LB with 0.25% arabinose after introduction of second *pmrB* alleles into PA14 and *pmrB*$_{L172del}$ backgrounds. The fold-change relative to the background strain is indicated in parentheses.**			
**Background strain**	**attTn7 Allele**	**POL7080 MIC (μg/mL)**	**Colistin MIC (μg/mL)**
PA14	---	0.10	0.88
PA14	*pmrB*$_{WT}$	0.20 (2)	0.88 (1)
PA14	*pmrB*$_{L172del}$	1.6 (16)	3.5 (4)
PA14	*pmrB*$_{G188S}$	0.40 (4)	1.8 (2)
PA14	*pmrB*$_{G188D}$	1.6 (16)	7.0 (8)
PA14-***pmrB**$_{L172del}$*	---	3.2	7.0
PA14-***pmrB**$_{L172del}$*	*pmrB*$_{WT}$	1.6 (0.5)	3.5 (0.5)

**Table S4: MICs of TAMRA-L27-11 against PA14 and the resistant PA14-*pmrB*$_{L172del}$ mutant.**	
	**TAMRA-L27-11**
**Strain**	**MIC (μg/mL) in MHB**
Wildtype PA14	14
PA14-***pmrB**$_{L172del}$*	>28

Introduction of *pmrB* alleles at the attTn7 chromosomal site

Copies of the wildtype *pmrB* allele, POL7080-resistant *pmrB* alleles L172del and G188S, and the colistin-resistant *pmrB* allele G188D(4) were introduced into PA14 at the neutral, naturally-evolved attTn7 chromosomal site using the mini-Tn7 system described previously in *P. aeruginosa*(5). When possible, the desired *pmrB* alleles were amplified from the genomic DNA of its corresponding mutant and inserted into the pUC18-derrived mini-Tn7 integration vector (with gentamicin cassette and AraC-*araBAD* promoter system) using standard PCR and Gibson Assembly protocols (New England Biolabs, Ipswich MA)(6). The G188D substitution was engineered into the wildtype PA14 *pmrB* mini-Tn7 integration vector using the Q5 Site-

Directed Mutagenesis Kit (New England Biolabs, Ipswich MA). Ultimately, each mini-Tn7 integration vector contained the desired *pmrB* allele (wildtype, L172del, G188S, or G188D) under control of the AraC-*araBAD* promoter(7), which encodes the *araC* repressor allowing for titratable *pmrB* gene expression in response to arabinose. After transformation into 10-beta *Escherichia coli* (New England Biolabs, Ipswich MA), bacterial conjugation was performed on cellulose membranes by spotting 20μL of a 1:2:2:2 mixture of the recipient strain (PA14 or PA14-$pmrB_{172del}$), 10-beta cells harboring the desired *pmrB* integration vector, and helper strains pRK2013 and pTNS3 (encoding the machinery necessary for pseudomonal plasmid uptake and subsequent integration at the attTn7 site). After mating at 37°C for 10 hours, cells were resuspended in 400μL LB and selected on LB agar containing 30μg/mL gentamicin and 15μg/mL irgasan, permitting growth of only PA14 cells containing the *pmrB* gene (and gentamicin cassette). Colonies were isolated and *pmrB* gene inserts were confirmed by fragment sizing after colony PCR. PCR products of the appropriate size were also recovered using the Gel Extraction Kit (Qiagen, Hilden Germany) and confirmed by sequencing.

RNAseq experiments

RNAseq experiments were carried out in biological triplicate in 384-well microplates (Nunc 384-well clear polystyrene plates, Thermo Fisher Scientific, Waltham MA). POL7001 was dissolved in DMSO at 200X the working concentration, and then diluted 1:100 into LB, yielding 2X drug solution in 1% (v/v) DMSO in LB. PA14 cultures were grown with shaking (37°C, 250rpm) to mid-log phase, and 30μL of cultures were mixed with equal volume of 2X POL7001 solution, yielding final conditions containing of $2x10^8$ CFU/mL PA14 with 0.2μg/mL POL7001 in 0.5% (v/v) DMSO in LB. Cells were treated at 37°C for 100 minutes without shaking in a humidity chamber. Samples were then mixed with 30μL of 3X RNAgem Blue Buffer (Zygem, Charlottesville VA), and chemically lysed by incubation in a thermocycler at 75°C for 10 minutes. Total RNA was then extracted using the Direct-zol kit (Zymo Research, Irvin CA), and RNA quality and quantity were analyzed using the RNA ScreenTape with the 2200 TapeStation (Agilent, Santa Clara CA). RNA-seq libraries were prepared using the RNA TagSeq protocol previously described(8), and samples were sequenced samples on a NextSeq instrument (Illumina, San Diego CA). Transcriptional data were analyzed using the Burrows-Wheeler Aligner(9) for alignment and DESeq2(10) to determine genes differentially expressed.

qRT-PCR experiments

qRT-PCR experiments were carried out in biological triplicate in a Nunc 96-well 2mL Deep Well Block (Thermo Fisher Scientific, Waltham MA). POL7080 was dissolved in DMSO at 200X the working concentration, and then diluted 1:100 into LB, yielding 2X drug solution in 1% (v/v) DMSO in LB. PA14 and resistant PA14-$pmrB_{L172del}$ cultures were grown with shaking (37°C, 250rpm) to mid-log phase in LB. 125μL of mid-log cultures were mixed with equal volume of either 2X POL7080 solution or 1% DMSO vehicle control, yielding final conditions containing of $2x10^8$ CFU/mL bacteria with 0.2μg/mL or 0μg/mL POL7080 in 0.5% (v/v) DMSO in LB. Cells were treated at 37°C for 100 minutes without shaking in a humidity chamber. Samples were then pelleted by centrifugation (5,000g, 10 minutes), and 150μL of supernatant was discarded. After resuspension in the remaining supernatant, 90μL of culture were transferred to an Axygen 96-well PCR plate (Corning Incorporated, Corning NY), containing 10μL of 10X RNAgem Blue Buffer (Zygem, Charlottesville VA). Chemically lysis was performed by incubation in a thermocycler at 75°C for 10 minutes. Total RNA was then extracted using the Direct-zol kit (Zymo Research, Irvin CA), and RNA quality and quantity were analyzed using the RNA ScreenTape with the 2200 TapeStation (Agilent, Santa Clara CA). Complementary DNA (cDNA) libraries were generated using the qScript cDNA synthesis kit (QuantaBio, Beverly MA). All qRT-PCR reactions were carried with the ViiA 7 Real-Time PCR system (Applied Biosystems, Foster City CA). Primer pairs were designed for *pmrA*, *arnB*, *pagL* and *rpoD* genes and purchased from Integrated DNA Technologies (**Table S5**). cDNA, primer pairs, and iTaq Universal SYBR Green Supermix (Bio-Rad Laboratories) were mixed according to the iTaq Universal SYBR Green Supermix protocol. 10μL of PCR reaction mixtures were transferred to 384-well thin-wall hard-shell PCR plates (Bio-Rad Laboratories, Hercules CA). Cycle conditions were based on the iTaq SYBR Green mix protocol for the BIO-RAD CFX384 system as follows: polymerase activation and DNA denaturation at 95ºC for 20 seconds for cDNA, or 5 minutes for genomic DNA; 40 cycles for amplification (denaturation at 95ºC for 5 seconds, followed by annealing at 63ºC for 30 seconds, followed by extension at 72ºC for 2 minutes). A series of seven 10-fold dilutions of PA14 control genomic DNA (initial stock at 40ng/μL) were performed, and standard curves were generated as previously described to determine the conversion factor of DNA copies per PCR reaction, assuming 1ng of PA14 genomic DNA equaled $1.4x10^5$ DNA copies(11, 12). For analysis, gene transcript levels were normalized to those of the housekeeping *rpoD*

gene, and subsequently normalized by untreated PA14 transcript levels to determine fold-change in gene expression relative to untreated PA14.

**Table S5: Optimal qRT-PCR primer pairs for genes *pmrA*, *arnB*, *pagL*, and *rpoD*.**		
**Gene**	**Forward Primer**	**Reverse Primer**
pmrA	AAGGCGATACCGTGGAATG	CAGGTTGCGCAGGATGT
arnB	CTGGCACCTGTTCATCCTG	AGGTGACTGGCGATGAAATG
pagL	GATGCGGGCTACACCTATTG	GCCTCGATGAATGGCTTGAT
rpoD	GATTTCCATCGCCAAGAAGT	CACGACGGTATTCGAACTTG

Synthesis of TAMRA-modified cyclic peptide L27-11 (TAMRA-L27-11)

Synthesis of parent peptide

The parent cyclic peptide L27-11 was synthesized mostly as previously described(13). The uncyclized linear peptide was prepared by a combination of batch synthesis and manual flow peptide synthesis (N^{α}-Fmoc-protected amino acids from Chem-Impex International)(14). Except where specified, all reagents in this procedure were purchased from Sigma-Aldrich. In batch, Fmoc-L-Pro-OH (1mmol) was coupled to 150 mg 2-chlorotrityl chloride polystyrene resin (200–400 mesh, loading: 1.14mmol/g, Chem-Impex International) in the presence of diisopropylethylamine (DIEA, 0.3mL) in dichloromethane (DCM, 2.5mL). Manual flow peptide synthesis followed the standard 3-minute cycle at 60°C previously described. Initial deprotection of N^{α}-Fmoc was performed with 6.6mL 20% (v/v) piperidine in N,N-dimethylformamide (DMF) delivered at 20mL/min over 20 seconds. Subsequent coupling was performed by delivering 1mmol Fmoc-Xaa dissolved in 2.5mL of 0.4M 2-(7-Aza-1H-benzotriazole-1-yl)-1,1,3,3-tetramethyluronium hexafluorophosphate (HATU, Chem-Impex International) in DMF and 0.5mL DIEA. Between coupling and deprotection steps, resin was washed for 1 minute by delivering 20mL of DMF at 20mL/min. Final amino acid coupling was performed in batch. Fmoc-N^{ε}-azide-L-Lysine-OH (1mmol) was dissolved in 2.5 mL of 0.4M HATU in DMF and 0.5mL DIEA, and added to resin. Subsequent Fmoc deprotection was performed by addition of 20% (v/v) piperidine in DMF (5mL). Cleavage from resin was performed via addition of 1% (v/v) TFA in DCM for 5 minutes. The eluate was transferred and neutralized with 1mL DIEA. Cleavage and neutralization was performed twice. Solvent was then removed under high vacuum. To cyclize, the resulting side-chain protected linear peptide was resuspended in DMF (85mL), and HATU (1mmol, 6 eq.), 1-hydroxy-7-azabenzotriazole (HOAt, 1mmol, 6 eq., Chem-Impex International), and 2,4,6-collidine (2.6mmol, 15 eq.) were added. The reaction was stirred for 72

hours, and DMF was then removed under high vacuum. Crude cyclic peptide was resuspended in DCM (20mL) and washed with 10% (v/v) acetonitrile (MeCN) in water containing 0.1% trifluoroacetic acid (TFA) twice. DCM fraction was transferred to a new vessel and DCM was removed under high vacuum. Crude cyclic peptide was then subjected to global deprotection via resuspension in TFA/thioanisole/water/phenol/1,2-ethanedithiol (82.5:5:5:5:2.5 v/v, 5mL) and incubation at room temperature for 2 hours. Deprotected peptide was precipitated from cleavage solution via trituration with ice cold diisopropyl ether. Crude cyclic peptide was washed twice with ice cold diisopropyl ether, dried, and conjugated to tetramethyl rhodamine (TAMRA).

Fluorophore conjugation and purification of TAMRA-L27-11

Crude parent peptide was conjugated to TAMRA using strain-promoted copper-free azide-alkyne click chemistry. Azide-containing parent peptide L27-11 (3mg) was resuspended in 1X phosphate buffered saline (PBS), and dibenzocyclooctyne-PEG4-TAMRA (3 mg, 2 eq., Sigma-Aldrich) resuspended in 5% (v/v) DMSO in 1X PBS was added to peptide, and mixture was incubated at room temperature for 2 hours. Reaction was then purified by preparative RP-HPLC (Agilent Zorbax SB C3 column: 9.4 x 250mm, 5μm) with a gradient of 5-55% MeCN in water with 0.1% TFA, run at 0.5% B/min. Pure fractions were pooled and lyophilized, and final purity was confirmed by LC-MS (**Figure S1**).

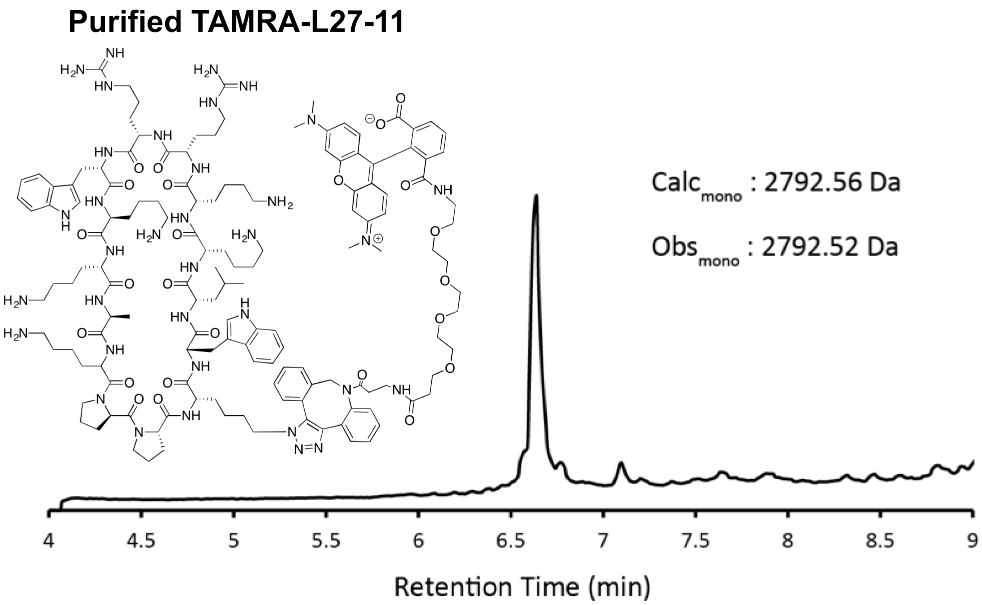

Figure S1. Chemical structure and LC-MS analysis of RP-HPLC purified TAMRA-modified cyclic peptide L27-11. Calculated and observed masses are monoisotopic, with total ion chromatogram shown.

Confocal Microscopy

PA14 and PA14-$pmrB_{L172del}$ cultures were grown with shaking (37°C, 250rpm) to mid-log phase in LB. TAMRA-L27-11 was dissolved in DMSO at 200X the working concentration, and then diluted 1:100 into LB, yielding 2X TAMRA-L27-11 solution in LB with 1% (v/v) DMSO. 500μL of mid-log bacteria were mixed in culture tubes with equal volume of TAMRA-L27-11 to yield a final bacterial concentration of $1x10^8$ CFU/mL in 1.4μg/mL TAMRA-L27-11 in LB with 0.5% (v/v) DMSO. After incubation with shaking (37°C, 250rpm) in the dark for 120 minutes, cells were pelleted and twice washed with PBS. All wash steps were carried out at room temperature by resuspension in 500μL PBS, centrifugation (4000g, 10 minutes), and careful removal of the supernatant without pellet disruption. Cells were next fixed by treatment with 4% (v/v) paraformaldehyde in PBS in the dark for 30 minutes on ice. Cells were washed once with PBS, resuspended in 100μL of 100μM 4′,6-diamidino-2-phenylindole (DAPI) dissolved in PBS, and incubated at room temperature for 20 minutes in the dark. Cells were again washed and resuspended in 20μL PBS. On clean microscope slides, 1μL of fixed cells were mixed with 5μL of ProLong Gold Antifade Reagent (Invitrogen, Carlsbad CA). Cover slips were applied and the samples were allowed to dry in the dark at room temperature for at least 24 hours. Blue and red-field confocal microscopy images were collected using the Zeiss Confocal Microscope. For analysis, the average intensities of about 20 cells were quantified using ImageJ software to calculate mean DAPI and TAMRA fluorescence intensities of PA14 and PA14-$pmrB_{L172del}$ after TAMRA-L27-11 treatment.

Supplemental References:

1. Winsor GL, Griffiths EJ, Lo R, Dhillon BK, Shay JA, Brinkman FS. 2016. Enhanced annotations and features for comparing thousands of Pseudomonas genomes in the Pseudomonas genome database. Nucleic Acids Res 44:D646-53.
2. Walker BJ, Abeel T, Shea T, Priest M, Abouelliel A, Sakthikumar S, Cuomo CA, Zeng Q, Wortman J, Young SK, Earl AM. 2014. Pilon: an integrated tool for comprehensive microbial variant detection and genome assembly improvement. PLoS One 9:e112963.
3. Wiegand I, Hilpert K, Hancock RE. 2008. Agar and broth dilution methods to determine the minimal inhibitory concentration (MIC) of antimicrobial substances. Nat Protoc 3:163-75.
4. Moskowitz SM, Brannon MK, Dasgupta N, Pier M, Sgambati N, Miller AK, Selgrade SE, Miller SI, Denton M, Conway SP, Johansen HK, Hoiby N. 2012. PmrB mutations promote polymyxin resistance of Pseudomonas aeruginosa isolated from colistin-treated cystic fibrosis patients. Antimicrob Agents Chemother 56:1019-30.
5. Choi KH, Schweizer HP. 2006. mini-Tn7 insertion in bacteria with single attTn7 sites: example Pseudomonas aeruginosa. Nat Protoc 1:153-61.
6. Gibson DG, Young L, Chuang RY, Venter JC, Hutchison CA, 3rd, Smith HO. 2009. Enzymatic assembly of DNA molecules up to several hundred kilobases. Nat Methods 6:343-5.
7. Wycuff DR, Matthews KS. 2000. Generation of an AraC-araBAD promoter-regulated T7 expression system. Anal Biochem 277:67-73.
8. Shishkin AA, Giannoukos G, Kucukural A, Ciulla D, Busby M, Surka C, Chen J, Bhattacharyya RP, Rudy RF, Patel MM, Novod N, Hung DT, Gnirke A, Garber M, Guttman M, Livny J. 2015. Simultaneous generation of many RNA-seq libraries in a single reaction. Nat Methods 12:323-5.
9. Li H, Durbin R. 2009. Fast and accurate short read alignment with Burrows-Wheeler transform. Bioinformatics 25:1754-60.
10. Love MI, Huber W, Anders S. 2014. Moderated estimation of fold change and dispersion for RNA-seq data with DESeq2. Genome Biol 15:550.
11. Nolan T, Hands RE, Bustin SA. 2006. Quantification of mRNA using real-time RT-PCR. Nat Protoc 1:1559-82.
12. Larionov A, Krause A, Miller W. 2005. A standard curve based method for relative real time PCR data processing. BMC Bioinformatics 6:62.
13. Srinivas N, Jetter P, Ueberbacher BJ, Werneburg M, Zerbe K, Steinmann J, Van der Meijden B, Bernardini F, Lederer A, Dias RL, Misson PE, Henze H, Zumbrunn J, Gombert FO, Obrecht D, Hunziker P, Schauer S, Ziegler U, Kach A, Eberl L, Riedel K, DeMarco SJ, Robinson JA. 2010. Peptidomimetic antibiotics target outer-membrane biogenesis in Pseudomonas aeruginosa. Science 327:1010-3.
14. Simon MD, Heider PL, Adamo A, Vinogradov AA, Mong SK, Li X, Berger T, Policarpo RL, Zhang C, Zou Y, Liao X, Spokoyny AM, Jensen KF, Pentelute BL. 2014. Rapid flow-based peptide synthesis. Chembiochem 15:713-20.
